# Effects of diaphragmatic control on multiparametric analysis of the sniff nasal inspiratory pressure test and inspiratory muscle activity in healthy subjects

**DOI:** 10.1371/journal.pone.0253132

**Published:** 2021-07-22

**Authors:** Kadja Benício, Vanessa R. Resqueti, Fernando A. L. Dias, Francesca Pennati, Andrea Aliverti, Jéssica Danielle Medeiros da Fonseca, Guilherme A. F. Fregonezi

**Affiliations:** 1 PneumoCardioVascular Lab/HUOL, Hospital Universitário Onofre Lopes, Empresa Brasileira de Serviços Hospitalares and Departamento de Fisioterapia Universidade Federal do Rio Grande do Norte, Natal, Brazil; 2 Laboratório de Inovação Tecnológica em Reabilitação, Departamento de Fisioterapia, Universidade Federal do Rio Grande do Norte, Natal, Brazil; 3 Departamento de Fisiologia, Universidade Federal do Paraná (UFPR), Curitiba, PR, Brazil; 4 Dipartimento di Elettronica, Informazione e Bioingegneria, Politecnico di Milano, Milan, Italy; University of Brasilia, BRAZIL

## Abstract

**Background:**

We investigated the influence of diaphragmatic activation control (diaphC) on the relaxation rate, contractile properties and electrical activity of the inspiratory muscles of healthy subjects. Assessments were performed non-invasively using the sniff inspiratory pressure test (SNIP) and surface electromyography, respectively.

**Methods:**

Twenty-two subjects (10 men and 12 women) performed 10 sniff maneuvers in two different days: with and without diaphC instructions. For the SNIP test with diaphC, the subjects were instructed to perform intense activation of the diaphragm. The tests with the best SNIP values were used for analysis.

**Results:**

The maneuver with diaphC when compared to the maneuver without diaphC exhibited significant lower values for: SNIP (p <0.01), maximum relaxation rate (MRR) (p <0.01), maximum rate of pressure development (MRPD) (p <0.01), contraction times (CT) (p = 0.02) and electrical activity of the sternocleidomastoid (SCM) (p <0.01), scalene (SCL) (p = 0.01) and intercostal (CI) (p = 0.03) muscles. In addition, the decay constant (tau, τ) and relaxation time (½ RT) did not present any changes.

**Conclusion:**

The diaphragmatic control performed during the SNIP test influences the inspiratory pressure and the contractile properties of inspiratory muscles. This occurs due to changes in the pattern of muscle recruitment, which change force velocity characteristics of the test. Thus, instruction on diaphC should be encouraged for better performance of the SNIP test and for evaluation targeting the diaphragm muscle activity.

## Introduction

The measurement of respiratory muscle strength has been used as an index for early detection of pulmonary dysfunction, as well as a parameter to assess the progression of diseases that cause respiratory muscle strength impairment. Additionally, it provides prognostic and predictive information on patient survival [1, 2]. In clinical practice, the methods commonly used for respiratory muscle assessment measure the Maximum Expiratory Pressure (MEP) and Maximum Inspiratory Pressure (MIP), the latter being complemented by the Sniff Nasal Inspiratory Pressure (SNIP). The SNIP is a non-invasive maneuver in which the patient performs an inspiratory effort, via one nostril, quickly and strongly. The sniff is a maneuver considered more physiological and easier to understand and execute, especially for children and people with neuromuscular disorders [3, 4].

The diaphragm is the largest and the main muscle involved in breathing. Over the years, transdiaphragmatic pressure (PDI) has been described as the most accurate measure to assess the strength of the diaphragm muscle [5]. This variable was studied by Miller et al. [6] who compared the PDIs obtained by MIP to the ones obtained by sniff. The results showed that PDI derived from sniff was greater than from MIP, and the authors suggested this was result of the diaphragm muscle being activated in greater intensity during the sniff maneuver. Thus, the sniff maneuver was considered a tool to detect diaphragmatic muscle weakness [6, 7].

The study of the SNIP test kinetics is suggested by the literature as a predictor of fatigue of the inspiratory muscles. This is represented by the relaxation rates described as maximum relaxation rate (MRR), half-relaxation time (½ RT) and constant curve of decay (τ, tau) after a maximum voluntary contraction, in addition to the contractile properties of the diaphragm such as maximum rate of pressure development (MRPD) and contraction time (CT) [8].

Ventilation is a complex process involving several structures and systems. The literature is controversial on the evaluation of respiratory muscles, especially regarding the most common tests used in a clinical environment. Occasionally the shortage of details to describe clinical tests, such as the SNIP test, can lead to methodological errors and misinterpretation of results. The American Thoracic Society / European Respiratory Society (ATS / ERS) guidelines in 2002 [9] states that "during the sniffing maneuver, patients are encouraged to make maximum efforts starting from relaxed end-expiration", stressing that "detailed instruction on how to perform the maneuver is not necessary, and may be counterproductive". The updated guideline by the ERS in 2019 [10] is even more evasive about this evaluation, limiting the instructions to: "the test is performed at FRC and the subject is instructed to sniff quickly and deeply". These guidelines do not elaborate that stimulation and a more detailed explanation or instruction to perform the sniff maneuver is an important way to obtain an effective test result, unlike the recommendations for MIP and MEP assessment. Although considered a more physiological maneuver, an individual can perform a sniff in an inadequate way when not properly instructed and stimulated. Thus, we hypothesize that individuals poorly instructed can make inadequate sniffs by recruiting more respiratory accessory muscles and therefore obtaining overestimated test results.

To our knowledge, the study carried out by Benício et al. [11] is the only one that proposed to evaluate the influence of diaphragmatic control (diaphC) during the SNIP test. It reports that diaphC maneuvers generate lower SNIP values, possibly due to a lower recruitment of accessory respiratory muscles, in addition to generating tests with technical standardization. As a result, the characteristics of sniff maneuvers without diaphC are different, which could determine errors in the test values. Therefore, it is necessary to investigate the importance of encouraging diaphragmatic contraction during the SNIP test in order to clarify the technical procedures for performing the maneuver.

The present study aims to evaluate the influence of diaphragmatic control on sniff nasal inspiratory pressure (1), relaxation rates and contractile properties of inspiratory muscles (2) and pattern of respiratory muscle activity (3) in healthy subjects.

## Methods

### Type of study and subjects

This is a quasi-experimental pre-post intervention study and was carried out at the PneumoCardioVascular Lab at the Universidade Federal do Rio Grande do Norte / Empresa Brasileira de Serviços Hospitalares (UFRN / EBSERH). Twenty-two self-reported healthy subjects of both genders, aged between 18 and 30 years were investigated. Participants were recruited by convenience in the University Community, city of Natal / RN between the period of August to November 2017. The inclusion criteria adopted were: body mass index (BMI) classified between 20–29.9 kg / m^2^, absence of history of cardiovascular, neurological, pulmonary diseases or diagnosis of deviated septum, no history of smoking; and presenting the spirometric variables of forced vital capacity (FVC) and forced expiratory volume ratio in the first second by the forced vital capacity (FEV_1_ / FVC) greater than 80% and 85% of the predicted value [12], respectively. We excluded subjects that failed to perform the tests/protocol, presented irregularities during data analysis or voluntarily requested their removal from the study. All study procedures were performed in accordance with the Declaration of Helsinki and National Research Ethics Commission resolution number 466/2012. Written informed consent was obtained and signed from all participants, and the study was approved by the Research Ethics Committee of Hospital Universitario Onofre Lopes (opinion 1.252.028 / 2015).

### Study design

The subjects were initially submitted to anamnesis, clinical, spirometric and respiratory muscle strength assessment. The SNIP evaluation protocol was performed in two different days with an interval of 48 hours, in order to minimize the learning effect. Assessments on both days were conducted by the same evaluator and the maneuvers were carefully demonstrated prior to commencement of the test, subsequently subjects were asked to repeat the maneuver for the purpose of familiarization [13, 14]. The SNIP assessments were carried out concomitantly with measurement of respiratory muscle activation using surface electromyography (sEMG). On day 1, after the clinical assessments, subjects had a resting period of 30 minutes before initiating the SNIP protocol. They were given instructions to perform a maximal effort starting from relaxed end-expiration (Functional residual capacity—FRC) following the guidelines of the ATS / ERS [10]. Participants performed ten sniff maneuvers without any instruction on diaphragmatic control (without diaphC). On day 2, the subjects were initially trained to breathe with a slow diaphragmatic breathing pattern. They were instructed to inhale deeply through the nose, while simultaneously moving the abdominal wall outward. A period of 5 to 10 minutes of training was established to confirm patients could execute the pattern correctly. Success was assessed visually, with maneuvers considered satisfactory when there was a clear increase in abdominal volume during inspiration [15]. After training the diaphragmatic breathing pattern, subjects were asked to move the abdomen outward repeatedly in a ballistic manner to familiarize themselves with the speed and strength required to perform the maneuver. There was a resting period of 30 minutes after training and before initiating the SNIP protocol to avoid muscle fatigue. Finally, participants performed ten sniff maneuvers associated to abdominal movement (diaphC). The same recommendations as day 1 were given to perform the sniff, with the additional emphasis on diaphragmatic control during its execution.

### Lung function

Spirometry was assessed using the KoKo DigiDoser® spirometer (Longmont, USA). The evaluations were carried out following the ATS / ERS [10] acceptability and reproducibility criteria and their reference values according to the values predicted for Brazilian adults [12]. Respiratory muscle strength was inferred by measuring the maximum inspiratory pressure (MIP), maximum expiratory pressure (MEP) and sniff nasal inspiratory pressure (SNIP) using the digital manovacuometer (NEPEB-LabCare / UFMG, Belo Horizonte—MG, Brazil). The evaluations were carried out according to the ATS / ERS [10] acceptability and reproducibility criteria. The MIP was measured with subjects seated in a chair with back support and using a nose clip to prevent air leakage during inspiration. They were instructed to perform a maximum expiration near residual volume (RV), followed by a deep inspiration against an occluded airway. The MEP was measured with the subject remaining in the same position as described above and performed a maximum expiration starting from total lung capacity (TLC) against the occluded airway. During the expiratory effort, the evaluator applied digital support to the cheeks in order to prevent air leakage. For MIP and MEP, the reference values previously published by Neder et al. [16] were used respecting the following criteria: presenting at least 3 reproducible and acceptable tests, the last maneuver cannot be the highest value of the series and the variability between the two best readings cannot exceed 10%. For the SNIP test, the subjects were sitting in a chair with back and arm support, with a plug inserted in the nasal orifice, while the other nostril remained free. They were instructed to breathe normally and to perform a sniff with maximum effort starting from FRC. Each subject performed 10 maneuvers, respecting a 1-minute rest between them, and the maneuver with the highest peak pressure was selected. The reference values of Araújo et al. [17] were used. For all pulmonary function variables, absolute values and the percentage of predicted values were used for analysis.

### Surface electromyography

**Fig 1:**
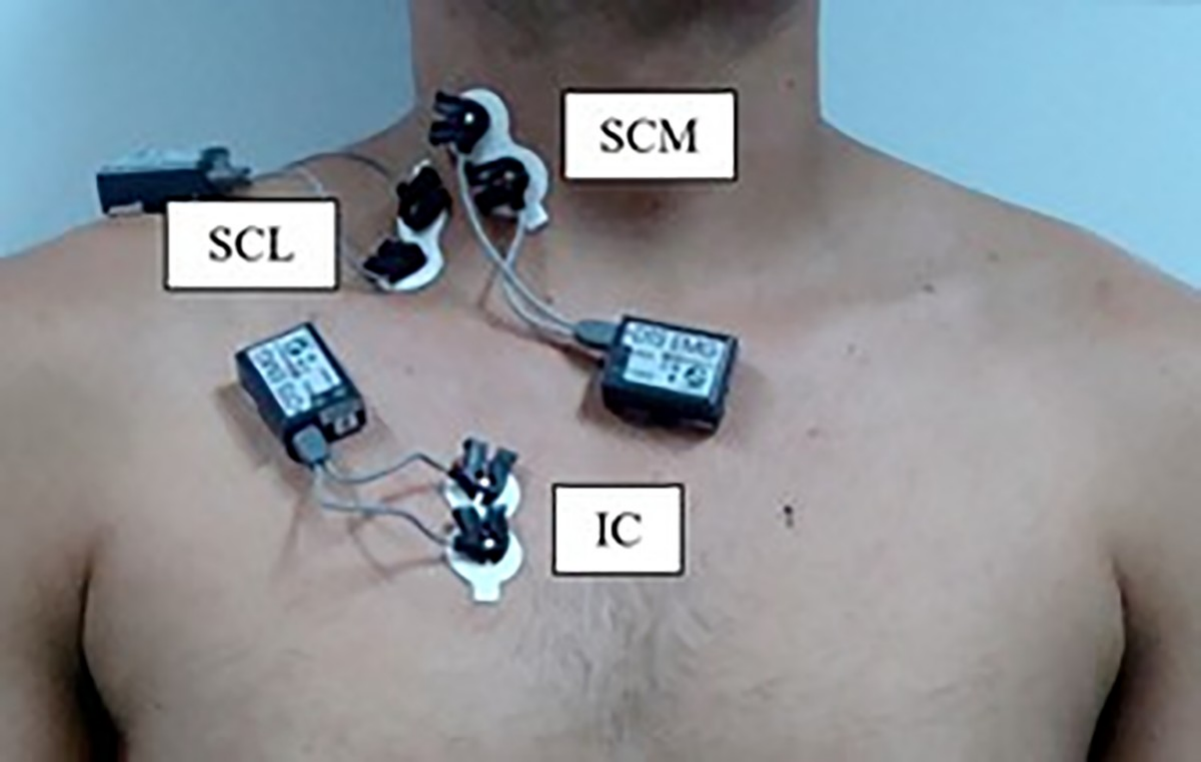
Representation of the electromyography electrodes positioning on SCL, SCM and IC muscles.

Surface electromyography was performed following the recommendations of the International Society of Electrophysiology and Kinesiology (ISEK) [18]. Myoelectric signals were recorded using the TeleMyo DTS Desk Receiver® device (Noraxon USA Inc., Scottsdale, USA) and 4 wireless sensors (Clinical DTS, Noraxon USA Inc., Scottsdale, USA) with a 20-500Hz pass filter-band, 1000 gain, 16-bit resolution, and a common mode rejection rate greater than 120 dB. Bipolar double trace Ag / AgCl (Miotec, Porto Alegre, Brazil) passive surface self-adhesive electrodes were placed on these following muscles: scalene (SCL), at a distance of 5 centimeters from the sternoclavicular joint and 2 centimeters above this point [19]; sternocleidomastoid (SCM), in the lower third of the distance between the mastoid process and the sternoclavicular joint [20]; and in the parasternal portion of the intercostal muscle (IC) over the second intercostal space and 3 cm from the sternum [21]. All electrodes were placed on the right side of the body to minimize cardiac noise interference. Before placing the electrodes, the skin region was shaved and cleaned with alcohol to reduce impedance. The software used to capture, process and store the signals was the MR 3.2 (Noraxon. Inc., Scottsdale, USA). Raw data was analyzed by means of RMS (root mean square) and standardized from respiratory baseline values [4]. Fig 1 shows the position of electromyography electrodes on SCL, SCM and IC muscles.

### SNIP curve analysis

The SNIP assessments (without diaphC and with diaphC) were performed starting from Functional Residual Capacity (FRC). The subjects performed a maximum sniff at the end of a slow and relaxed exhalation with the mouth closed, responding to a verbal command of “pull”. The evaluation was considered complete when 10 acceptable maneuvers were performed without air leakage and respecting a one-minute rest interval between them [22]. At the end of the measurement, the highest SNIP value obtained, with a properly plotted graph, was used for data analysis. The criteria used to select the appropriate sniff maneuvers for analysis were: (1) peak pressure sustained for less than 50 milliseconds (ms) and without biphasic peak, (2) total sniff duration less than 500 ms and (3) shape of sniff pressure curve that presents a smooth decay [23].

The variables for the multiparametric analysis of the SNIP curve were calculated from the sniff maneuver trace. CT and ½ RT were calculated as the time to reach the peak sniff pressure and the half-time of the relaxation curve, respectively [24]. MRPD, expressed in cmH_2_O·ms^−1^, was calculated as the negative peak of the first derivative from the pressure-time curve [25], MRR, expressed in milliseconds^−1^, was defined as the positive peak of the first derivative from the pressure-time curve normalized by the peak pressure, in order to make contractions of different intensities comparable [26]. The time constant (τ, tau) was calculated by plotting the natural logarithm of pressure as a function of time. The lower 50–70% of the pressure decay curve follows a straight line [26], indicating that the pressure follows a monoexponential decay with a time constant τ (τ = 1 / slope). For the τ measure to be accepted, the correlation coefficient of the individual regression line (log(pressure) vs. time) should be ≥ 0.96 [27]. SNIP curves were analyzed by custom software developed in MatLab (MathWorksInc, Natick, MA, USA) by TBM lab at Politecnico di Milano. The graphical representation of the multiparametric analysis of the SNIP curve was published in a previous study this same group by Sarmento et al. [8] and is available in the S1 Fig.

### Statistical analysis

The sample size was established considering SNIP as the main variable. We analyzed five subjects using a hypothetical paired t test with mean and standard deviation (SD) for without diaphC (96 ± 16.7) and with diaphC (76.8 ± 22.8) groups. A sample of 14 subjects was estimated using an alpha error of 0.05 with bilateral distribution and a test power of 80%.

Data normality and distribution were verified using the Shapiro-Wilk test. The analyses between the different sniff maneuvers (without diaphC and with diaphC) were studied using the paired t test or Wilcoxon for parametric and non-parametric data, respectively. The GraphPad Prism 6.0 program (GraphPad Software, San Diego, USA) was used for data analysis. Study power (β) and effect size (ES) [28] were calculated and are detailed in the main study variables. The sample calculation, study power and effect size were analyzed using the G*Power software, version 3.1.9.2 (Kiel, Germany). A level of significance, p<0.05 with bilateral distribution was adopted for all statistical analyzes.

## Results

**Fig 2:**
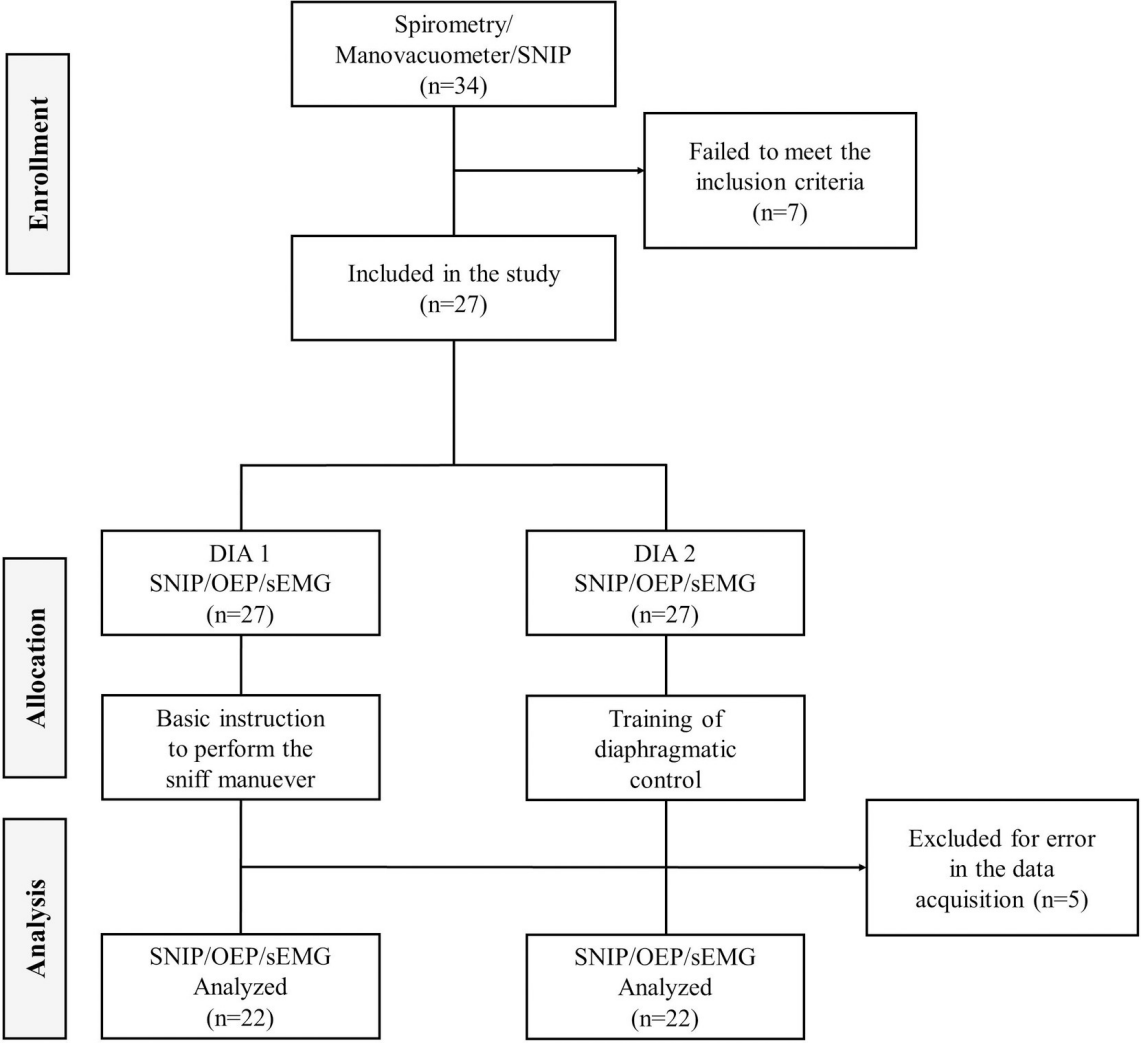
Study design.

Thirty-four subjects were screened, of which 27 were included in the study. Seven subjects failed to meet the inclusion criteria—five due to a FEV1 / FVC ratio less than 85% and two due to the presence of deviated septum. Additionally, five subjects were excluded after the complete evaluation—three due to the low quality of their electromyographic signals and two because it was not possible to obtain appropriate SNIP curves for graphic analysis (correlation coefficient of individual regression line ≤ 0.96). Resulting in a final sample of 22 subjects (12 female, 10 male). The study design is shown in Fig 2. The sample anthropometric characteristics, pulmonary function and respiratory muscle strength data are shown in Table 1.

**Table 1:** Sample description.

**Description**	
Subjects (n)	22
Gender F/M	12/10
Age, yrs	23.7 ± 2. 2
Weight (Kg)	65.5 ± 8.9
Height (m)	1.69 ± 0.9
BMI (Kg/m^2^)	22.8 ± 1.9
**Pulmonary function**	
FEV_1_ L	3.79 ± 0.73
% predicted	92.9 ± 9.6
FVC L	4.49 ± 0.94
% predicted	96. 8 ± 9.1
FEV_1_/FVC	0.85 ± 0.06
% predicted	96.2 ± 8.62
MIP cmH_2_O	121.5 ± 31.6
% predicted	105.7 ± 26.1
MEP cmH_2_O	118.7 ± 28.5
% predicted	98.1 ± 16.4

Values are mean ± SD. F: female; M: male; yrs: years; BMI: Body Mass Index; kg: kilograms; m: meters; FEV_1_: Forced Expiratory Volume in the first second; L: liters; FVC: Forced Vital Capacity; MIP: Maximum Inspiratory Pressure; cmH_2_O: centimeters of water; MEP: Maximum Expiratory Pressure; SNIP: Sniff Nasal Inspiratory Pressure.

**Fig 3:**
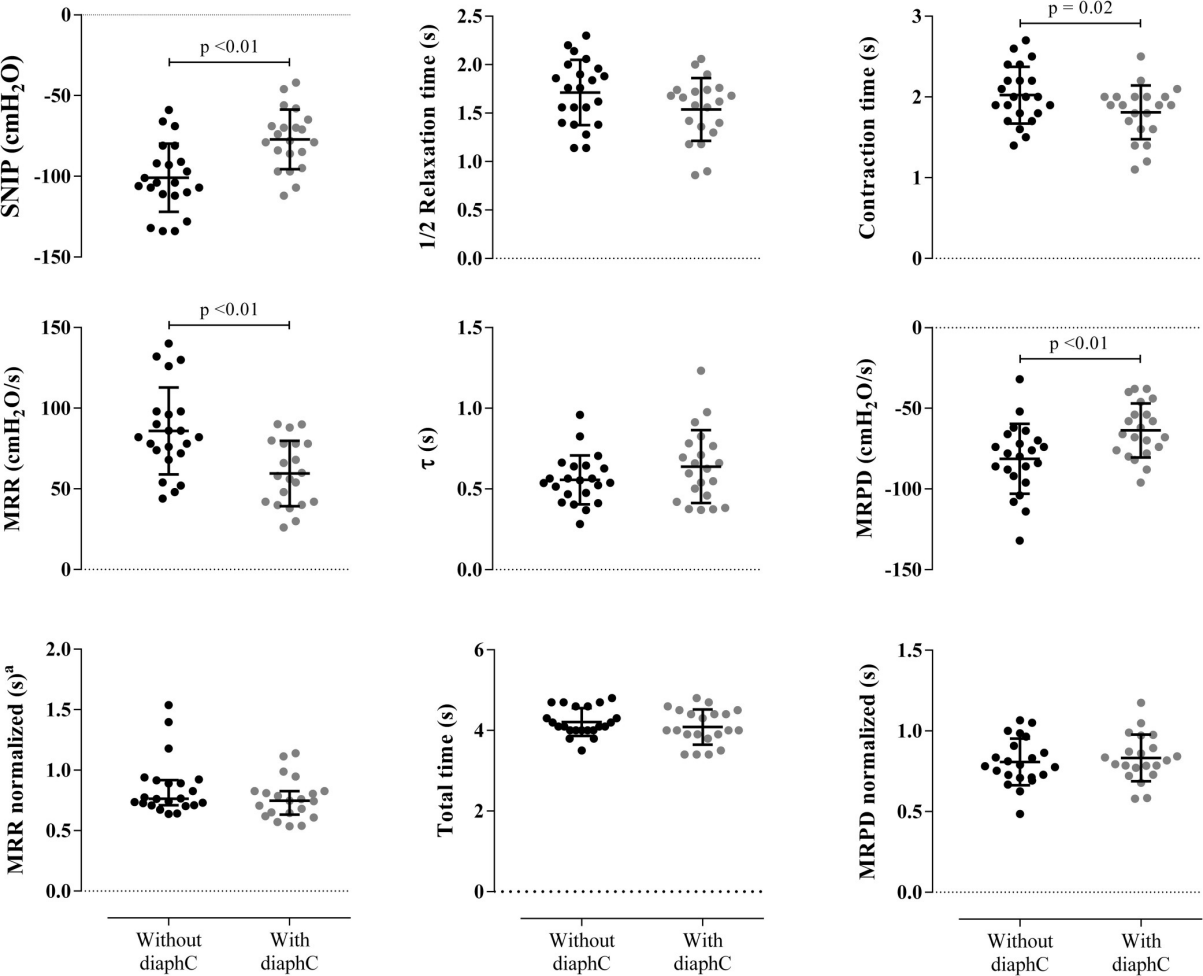
Multiparametric analysis. Data are shown as mean ± SD. Comparisons between maneuver with and without diaphC in the parameters obtained from the sniff nasal inspiratory pressure (SNIP) curve. MRR: maximum relaxation rate; MRPD: maximum rate of pressure development; CT: contraction time; ½ RT: half-relaxation time; τ: tau; cmH2O: centimeters of water; s: seconds.

Fig 3 shows the parameters extracted from the multiparametric analysis of the SNIP curve. A significant reduction in SNIP, MRR and MRPD was observed in the maneuvers performed with diaphC compared to without diaphC (p <0.001), however there was no statistical difference between MRR and MRPD after normalization (p = 0.82 and p = 0.31). The variables related to relaxation, τ and ½ RT, did not show differences between the maneuvers (p = 0.16 and p = 0.13, respectively). The CT was significantly higher in the diaphC maneuver (p = 0.02). The total time (T_TOT_) did not vary between maneuvers (p = 0.23).

**Fig 4:**
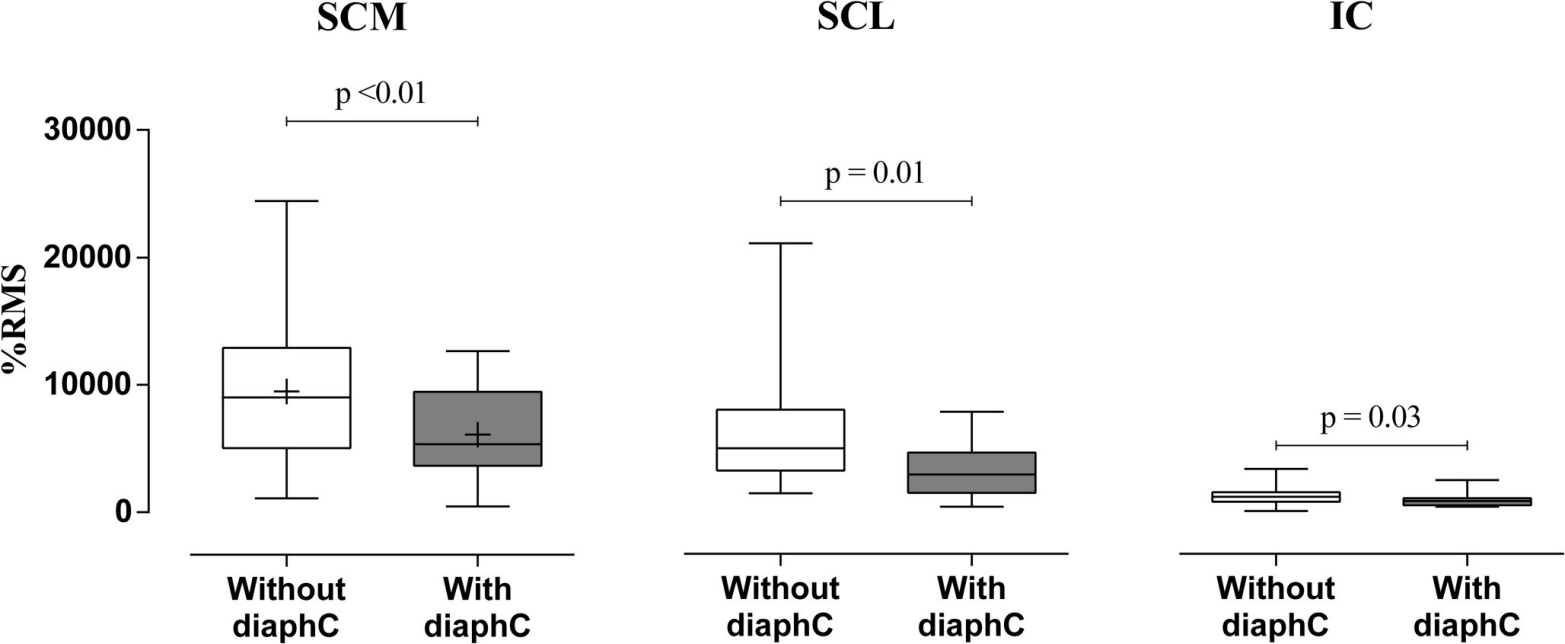
sEMG. Data are shown as median [25–75^th^ percentile]. Percentage of respiratory muscle activation during the maneuver with and without diaphC. SCM: sternocleidomastoid; SCL: scalene; IC: Intercostal; +, mean for parametric analysis.

The RMS values for the respiratory muscles were normalized and are shown in Fig 4. When performed the maneuver with diaphC the % RMS of the SCL, SCM and IC muscles (p = 0.01, p <0.001 and p = 0.03, respectively) showed lower values compared to the maneuver without diaphC.

**Fig 5:**
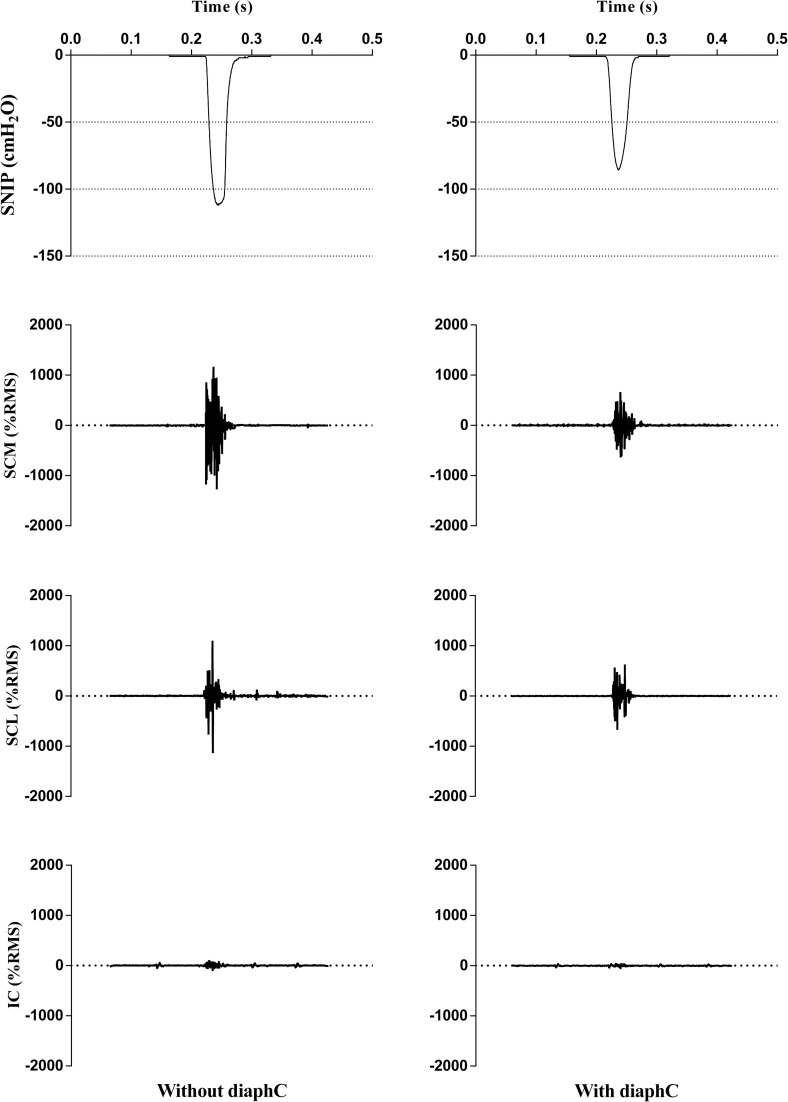
Scalar tracing. Scalar tracing of SNIP and electrical activity of scalene (SCL), sternocleidomastoid (SCM) and intercostal (IC) muscles, during the maneuver with and without diaphC.

Fig 5 shows the representation of the SNIP test kinetics concomitant with the capture of electromyographic activity of SCM, SCL and IC muscles during maneuvers with and without diaphC. It displays the pressure reached, the total time of the maneuver and the peak activity of the respiratory muscles assessed.

Table 2 reports the effect size and power test for comparisons between maneuver with and without diaphC in the parameters obtained from the sniff nasal inspiratory pressure (SNIP) curve. MRR: maximum relaxation rate; MRPD: maximum rate of pressure development; CT: contraction time; ½ RT: half-relaxation time; τ: tau and sEMG RMS values of SCM, SCL and IC muscles.

**Table 2:** Effect size and power test.

	Comparisons between maneuver with and without diaphC	
	Effect size Cohen’s dz	Power
SNIP (cmH_2_O)	1.68	<0.99
MRR (cmH_2_O/s)	0.97	0.99
MRR *normalized*	0.27	0.23
MRPD (cmH_2_O/s)	1.06	0.99
MRPD *normalized*	0.23	0.18
½ RT (s)	0.32	0.3
T_TOT_ (s)	0.26	0.21
CT (s)	0.54	0.68
τ (s)	0.14	0.09
SCM (%RMS)	0.64	0.71
SCL (%RMS)	0.59	0.63
IC (%RMS)	0.5	0.49

Comparisons between maneuvers with and without diaphC in the parameters obtained from the sniff nasal inspiratory pressure (SNIP) curve and respiratory muscle activation. MRR: maximum relaxation rate; MRPD: maximum rate of pressure development; ½ RT: half-relaxation time; T_TOT_: total time; CT: contraction time; τ: tau; cmH_2_O: centimeters of water; s: seconds; SCM: sternocleidomastoid; SCL: scalene; IC: Intercostal; %RMS: percentage of root mean square.

## Discussion

The aim of this study was to evaluate the influence of diaphragmatic activation control on the variables resulting from the sniff nasal inspiratory pressure test and inspiratory muscle activity. Additionally, we aimed to determine the technical standardization for performing the sniff maneuver in healthy subjects.

The main findings of this study show that the maneuver with diaphC compared to without diaphC presents: 1) significantly lower SNIP value, 2) reduced activity of SCM, SCL and IC muscles; 3) reduced absolute values of MRR and MRPD, since they are directly proportional to the pressure obtained (dP / dt), however values normalized by the peak pressure showed no different behavior, and 4) decreased contraction time.

Diaphragmatic activation control influences the SNIP by reducing its values. Which supports the hypothesis that SNIP values are lower when a ballistic contraction of the diaphragm muscle is performed during a sniff maneuver. This is a consequence of respiratory accessory muscles being less recruited and, therefore, the diaphragm is more targeted. This is based on the findings described by Benício et al. [11].

During a breathing cycle, several muscles act on the rib cage to facilitate breathing. The contraction of diaphragm is directly related to the inspiration phase, while its relaxation favors the basal expiration. The expiration occurs passively as a result of the lungs elastic recoil. During the measurement of maximum respiratory pressures, the subject performs a forced inspiration, in which the diaphragm and respiratory accessory muscles act together.

The assessment of maximum respiratory pressures represents the measurement of respiratory muscle strength and can be assessed by volitional or non-volitional tests. Volitional tests are simple, portable and inexpensive, however they rely on maximum voluntary neuromuscular activation [29] which can be considered a limitation. MIP is the volitional test commonly used for inspiratory muscle strength assessment and it is measured during the performance of a maximum forced inspiration against a pressure gauge using a mouthpiece. It is sometimes considered difficult to perform, which may result in lower values when there are air leaks, as well as in cases of lack of motivation or coordination by the subject being assessed [30]. Currently, the SNIP test has been used to complement the MIP on the assessment of inspiratory muscles. For such, the sniff maneuver is performed with the occlusion of one nostril against a pressure manometer. The sniff is considered easier to perform as it is a more natural maneuver [30], however, there is little data regarding standardization of the SNIP test and the assessment of muscle recruitment patterns during the test.

Verin et al. [31] studied how voluntarily changing muscle recruitment affects sniff esophageal (P_ES_), gastric (P_GA_) and transdiaphragmatic (P_DI_) pressures. They assessed 3 different types of sniff maneuvers: natural, diaphragmatic and extra diaphragmatic. The results showed that when performing natural sniff maneuvers the subjects vary their recruitment pattern between diaphragmatic and extra diaphragmatic. However, the performance of diaphragmatic sniff maneuvers presented higher values of transdiaphragmatic and gastric pressures compared to the other patterns. The authors emphasize the need for studies assessing the benefits of abdominal dislocation (i.e., diaphragmatic control) during the SNIP test. Similar to our results, the study by Benício et al. [11] exposed that performing diaphragmatic control during sniff maneuvers results in lower SNIP value when compared to maneuvers without diaphC. They associated this result to the likely difference in muscle recruitment during both maneuvers. These studies are, to our knowledge, the only ones that proposed to evaluate the influence of diaphragmatic control during the SNIP test and our results complement the authors’ interpretations.

The surface electromyography of the respiratory muscles evaluated during the SNIP test helps to clarify the results of this study. A reduction in the activity of respiratory accessory muscles was observed after training on diaphragmatic activation control (i.e., maneuver with diaphC) when compared to without diaphC. This indicates that despite the increase in transdiaphragmatic pressure in maneuvers with diaphC [31], reducing the action of accessory muscles reduces SNIP values.

Previous studies have assessed the electromyographic activity of respiratory muscles during sniff maneuvers [32, 33]. Nava et al. [33] evaluated 3 different maximal inspiratory maneuvers, which demonstrated that the sniff maneuver has a higher diaphragmatic activation pattern, represented by higher values of diaphragmatic pressure and electrical activity of the diaphragm muscle. Additionally, they reported that the recruitment pattern of the inspiratory muscles of the rib cage were similar during sniff and Müller maneuvers. Katagiri et al. [32] evaluated the activation of accessory muscles during the sniff maneuver and demonstrated the performance of the scalene muscle during low intensity sniff and additional activity of the sternocleidomastoid muscle in high intensity sniff.

Therefore, we can infer that maneuvers with diaphC present reduced SNIP values due to a decreased activity of the rib cage muscles, resulting in a more targeted expression of the diaphragm activity. In contrast, the maneuvers without diaphC recruit the respiratory accessory muscles more strongly and, therefore, the characteristics of the sniff maneuver are modified, which overestimates the test values.

The study of the SNIP test kinetics has been used as an indirect marker of muscle fatigue and inspiratory muscle overload and it is represented by the maximum relaxation rate (MRR). This occurred after Kyroussis et al. [34] reported that the MRR obtained non-invasively by performing a sniff maneuver reflects the value of the MRR measured at esophageal pressure curves.

Esau et al. [7, 35] suggested that the rate of decline in the PDI reflects the MRR, as the electrical activity of the diaphragm muscle ceases when the PDI decreases, therefore the beginning of the pressure drop coincides with the beginning of the diaphragm relaxation. MRR has been evaluated through analysis of the pressure curves of P_DI_ [27, 36], P_ES_ [37], P_ORAL_ [23, 38] and SNIP in several studies. All of these studies assumed that the variation of pressure reflects the changes in the diaphragm length-tension due to the coincidence between the interruption of diaphragm activity and the beginning of pressure decay. Several studies have shown that the slowdown in MRR indicates respiratory muscle fatigue, especially the diaphragm [23, 39].

Diaphragmatic control influenced the MRR reducing its values. However, when the MRR was normalized by the peak pressure obtained in the same maneuver, no difference was observed between sniff maneuvers with or without diaphC. The interpretation of MRR values in this scenario should be cautious, considering that its reduced values in diaphC maneuvers do not reflect muscle weakness, but rather a change in muscle recruitment—emphasizing the diaphragm more than other inspiratory muscles. This change promoted the reduction of SNIP and consequently MRR, as the latter is a measure directly proportional to the inspiratory pressure obtained. The normalization of MRR by peak pressure (MRRnorm = (dP / dt) / P_SNIP_ * 100) aims to exclude the effect of pressure oscillation amplitude [34], being the real representation of the relaxation rate.

Literature shows τ and ½ RT as complementary in the evaluation of relaxation kinetics. These variables also did not show variations in the sniff maneuvers performed with and without diaphC. This result was expected, since the experiment proposed to evaluate the influence of diaphragmatic control on sniff maneuvers and not respiratory muscle fatigue. Thus, we can understand that the execution of diaphragmatic control did not change the relaxation kinetics in the evaluated subjects.

The contractile properties of respiratory muscles (MRPD and CT) are still poorly studied and scarce in the literature. The MRPD represents the positive peak of the pressure derivative over time during the initial slope of the maximum respiratory pressure curve. Similar to the MRR, the MRPD is under the effect of variations in pressure amplitude and due to this fact, it also had its values normalized by peak pressure. Tzelepis, Kasas and McCool [40] observed that muscle training protocols increased lung function by analyzing the MRPD and showing that the increase in MRPD was directly proportional to the inspiratory pressure. Our results also followed this pattern, the MRPD values were higher in maneuvers without diaphC. However, when normalized, the MRPD did not differ, showing that muscle activation is equivalent in both maneuvers.

Muscle fatigue is observed by an increase in contraction time. This is explained by a greater number of motor units recruited in situations of muscle stress. Our results show that diaphC maneuvers presented lower CT values when compared to sniffs without diaphC as a result of the different muscular recruitment of each maneuver. DiaphC maneuvers recruit primarily the diaphragm muscle which reduces the total number of motor units recruited and consequently the CT.

Benício et al. [11] also question the technical characteristics of sniff maneuvers. The authors reported 40% of the maneuvers without diaphC exceeded the maneuver time recommended in the literature. This difference was not evidenced in the present study, since we adopted in our inclusion criteria the technical characteristics recommended in the literature [23] and therefore, none of the tests selected for evaluation presented a contraction time greater than 500 ms. Additionally, no differences were found in the total time between the maneuvers.

In summary, our results show that diaphragmatic activation control modifies the kinetics of the SNIP test. It emphasizes the action of the diaphragm muscle by reducing the action of rib cage muscles, without changing the relaxation properties and improving the test precision to study the function of the diaphragm. Thus, we suggest that interpretation of SNIP values depends on the purpose of the assessment. SNIP should be used as an indicator of global inspiratory muscle function when the maneuver without diaphC is adopted, emphasizing that inspiratory pressure values resulting from this maneuver overestimate the SNIP. Alternatively, maneuvers with diaphC should be prioritized in assessing muscle function directly related to the diaphragm.

The study showed limitations in its evaluation format. We understand that gender differences in contraction and relaxation properties, electrical activity of respiratory muscles and SNIP values may exist. Unfortunately, we did not perform this analysis due to the sample size. We strongly suggest that future studies consider this type of analysis. Another limitation was that evaluation and analysis of data was not blind, which increased the risk of bias in the study. However, we analyzed the power and effect size in order to strengthen our main results. The direct evaluation of the diaphragm muscle electrical activity would reinforce our results. However, we were unable to capture the activity of this muscle using sEMG and we could not use invasive resources for this measurement. Nonetheless, it is important to note that same conditions were applied in both assessments to allow for comparison. In addition, we emphasize that applying this technique can direct the results of the maneuver and assist with interpretation of its values.

## Conclusions

Encouraging diaphragmatic contraction changes the characteristics of the SNIP test. The maneuvers without diaphC recruit respiratory accessory muscles more strongly and, therefore, the characteristics of the sniff maneuver are modified, which determines higher test values.

We conclude that for a more effective assessment of diaphragmatic strength, the diaphragmatic control should be encouraged during the SNIP test. The maneuver must be explained in detail and demonstrated by the examiner, since it allows for a more accurate measurement within the standards described in literature.

## Supporting information

S1 Fig Representative tracings of the sniff nasal inspiratory pressure (SNIP) test and its parameters. (A) Tracings of SNIP change; peak sniff pressure (P_sniff_); time to reach P_sniff_, contraction time (CT); and half-time of the relaxation curve (1/2RT). (B) Derivative signal of sniff pressure (dPressure_sniff_/dT = cmH_2_O/ms); negative peak dP_sniff_/dT, maximum rate of pressure development (MRPD) positive peak dP_sniff_/dT normalized by P_sniff_, maximum relaxation rate (MRR). (C) Decay portion of the sniff pressure plotted on semilog scale vs. time (ms). Linear black portion indicates a single exponential function with a time constant, τ = 1/slope. cmH_2_O, centimeters of water. This figure is republished from Sarmento et al. [8] under a CC BY license, with permission from Frontiers in Neurology, original copyright © 2018 Sarmento, Aliverti, Marques, Pennati, Dourado-Júnior, Fregonezi and Resqueti. This is an open-access article distributed under the terms of the Creative Commons Attribution License (CC BY). The use, distribution or reproduction in other forums is permitted, provided the original author(s) and the copyright owner are credited and that the original publication in this journal is cited, in accordance with accepted academic practice. No use, distribution or reproduction is permitted which does not comply with these terms.
(TIF)

S1 Data (RAR)
