# Supplementary figures and images for: Effects of diaphragmatic control on multiparametric analysis of the sniff nasal inspiratory pressure test and inspiratory muscle activity in healthy subjects
Source: PLoS One. 2021 Jul 22;16(7):e0253132. doi: 10.1371/journal.pone.0253132 (PMC8297810; doi:10.1371/journal.pone.0253132)

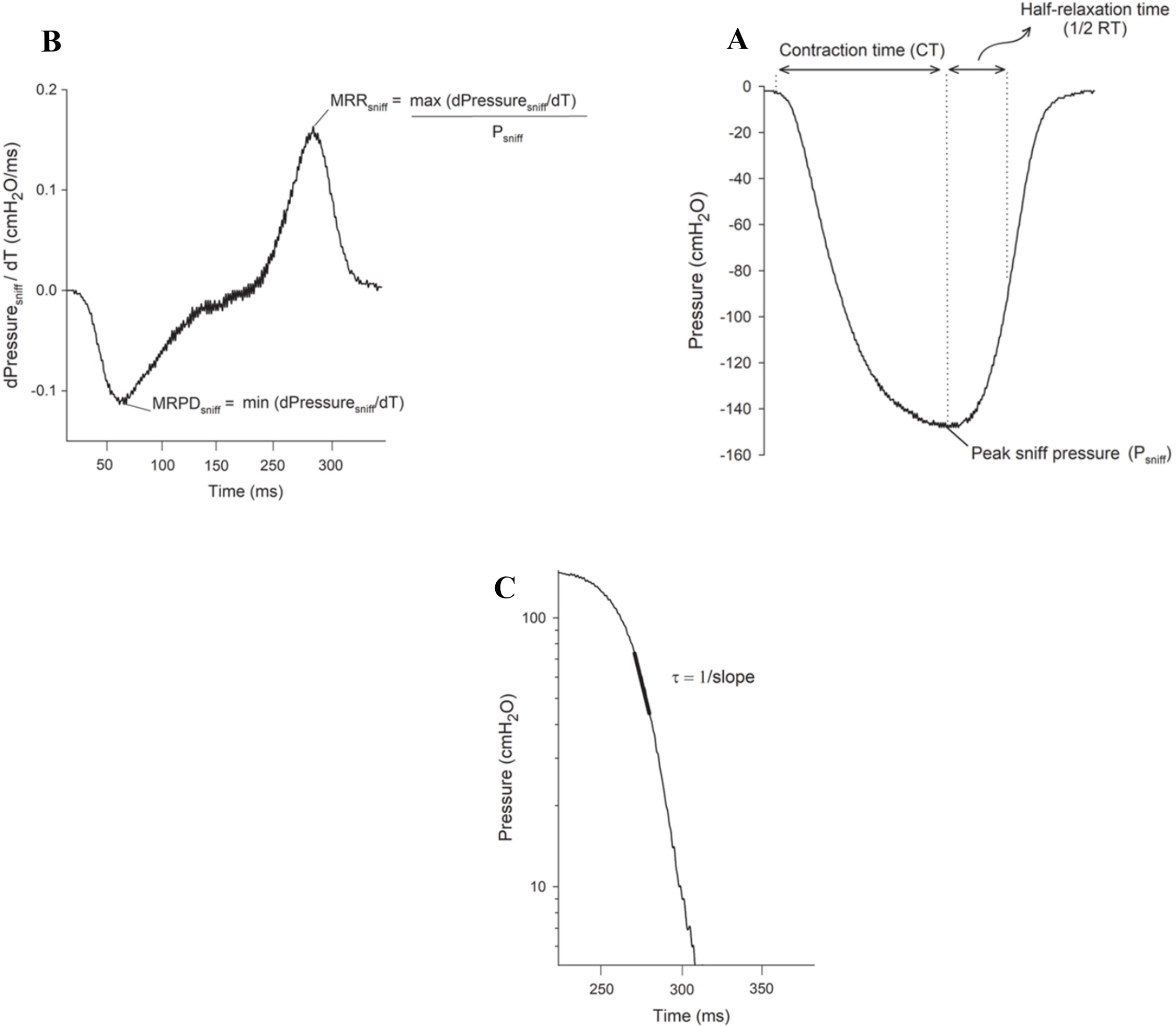

Supplement: S1 Fig — (A) Tracings of SNIP change; peak sniff pressure (Psniff); time to reach Psniff, contraction time (CT); and half-time of the relaxation curve (1/2RT). (B) Derivative signal of sniff pressure (dPressuresniff/dT = cmH2O/ms); negative peak dPsniff/dT, maximum rate of pressure development (MRPD) positive peak dPsniff/dT normalized by Psniff, maximum relaxation rate (MRR). (C) Decay portion of the sniff pressure plotted on semilog scale vs. time (ms). Linear black portion indicates a single exponential function with a time constant, τ = 1/slope. cmH2O, centimeters of water. This figure is republished from Sarmento et al. [8] under a CC BY license, with permission from Frontiers in Neurology, original copyright © 2018 Sarmento, Aliverti, Marques, Pennati, Dourado-Júnior, Fregonezi and Resqueti. This is an open-access article distributed under the terms of the Creative Commons Attribution License (CC BY). The use, distribution or reproduction in other forums is permitted, provided the original author(s) and the copyright owner are credited and that the original publication in this journal is cited, in accordance with accepted academic practice. No use, distribution or reproduction is permitted which does not comply with these terms. (TIF) [file pone.0253132.s001.tif]
